# Gut microbiota and cognitive decline: a scoping review of microbial mechanisms and adaptive responses in dementia

**DOI:** 10.3389/fnagi.2026.1782720

**Published:** 2026-03-31

**Authors:** Giovanni Luca Cipriano, Alessandro Grimaldi, Angela Marra, Angelo Quartarone, Giuseppa Maresca

**Affiliations:** IRCCS Centro Neurolesi “Bonino-Pulejo”, Messina, Italy

**Keywords:** Alzheimer’s disease, cognitive decline, dementia, dysbiosis, gut microbiota, gut-brain axis, neuroinflammation

## Abstract

Dementia is a progressive disease that results in a loss of mental capacity. Some of the most affected cognitive skills are memory, orientation, and language. These skills are also associated with behavioral shifts such as increased agitation and apathy, worsening the affected person’s quality of life. The most common type of dementia is Alzheimer’s disease, and it is especially concerning in older adults. Alzheimer’s is characterized by the formation of beta-amyloid plaques and neurofibrillary tangles that are made of hyperphosphorylated tau proteins. These plaques and tangles lead to inflammation in the central nervous system, damage to the connections between neurons, and overall degeneration of the nervous system. Newer studies have started to identify the gut microbiome and the gut-brain axis as components critical to the progression of neurodegenerative diseases. Dysbiosis, which is characterized by an imbalance or loss of microbial diversity in the gut, has been attributed to the worsening of neurodegenerative diseases. The gut microbiome has been shown to have a large impact on the brain and how it responds neurochemically. An imbalance in the gut microbiome has also been shown to lead a person to emotional and cognitive dysfunction. It has been shown that in dementia patients, there is also an associated intestinal dysbiosis and increased inflammation systemically and within the brain. Certain gut bacteria stimulate the production of pro- inflammatory cytokines and neuroinflammation, which is a defining characteristic of diseases associated with dementia. This review is focused on three main aspects in which dysbiosis is related to cognitive decline.

## 1 Introduction

Dementia is a progressive neurodegenerative disorder which is marked with a deterioration of cognitive functions such as memory, orientation, language, and executive functions as well as behavioral changes of agitation, apathy, and aggression which diminishes quality of life (Cerejeira et al., 2012). The most common type of dementia, Alzheimer’s disease (AD), is an increasingly important health issue, particularly with aging populations (Javaid et al., 2021). Neuropathologically, AD is marked by the presence of beta-amyloid (Aβ) plaques and intracellular neurofibrillary tangles of hyperphosphorylated tau, which causes neuroinflammation, synaptic loss, and neurodegeneration (Takahashi et al., 2017). Current evidence positions the gut-brain axis not merely as an associative factor, but as a critical modulator of neurodegenerative progression. This axis functions as a bidirectional communication system linking the gastrointestinal tract and the central nervous system, where neurological, immune, and metabolic pathways interact to maintain brain homeostasis (Loh et al., 2024). Alterations of the gut microbiota, known as dysbiosis, have been associated with the onset and progression of neurodegenerative disorders such as dementia (Zhao et al., 2017; Cattaneo et al., 2017; Kim and Jazwinski, 2019). Experimental studies indicate that the microbiota modulates the brain’s neurochemical response and that an imbalance in the gut flora can impair emotional and cognitive balance, contributing to the development of cognitive and behavioral symptoms typical of dementia (Cryan and O'Mahony, 2011). Intestinal dysbiosis has been observed in patients with dementia, including AD, and is believed to contribute to systemic and brain inflammation, promoting neuronal damage. Gut bacteria can modulate the production of pro-inflammatory cytokines, which trigger neuroinflammation—a key process in neurodegenerative diseases (Zhao et al., 2017; Vogt et al., 2017; Liang et al., 2018). Zhao et al. (2017) highlighted that dysbiosis in Alzheimer’s patients correlates with elevated levels of inflammatory markers in blood and brain. Moreover, the gut microbiota can influence the production of beta-amyloid, the protein that accumulates in the brains of people with AD. Many studies suggest that the gut microbiota can also influence behavior (Cryan and Dinan, 2012; Sgambato and O’Mahony, 2020), especially in patients with dementia, where symptoms such as depression, anxiety, aggressiveness, and apathy are common. Dysbiosis disrupts the enzymatic machinery required for the synthesis of key neuroactive precursors (e.g., tryptophan availability for serotonin) (Cryan and O'Mahony, 2011; Hoban et al., 2016; Maier et al., 2017), thereby directly impairing synaptic plasticity and emotional regulation, while its alteration can cause behavioral disturbances in patients with dementia. Neuroinflammation is a key factor in the development of dementia and other neurodegenerative diseases. The gut microbiome can influence this process through the production of pro-inflammatory molecules like cytokines, which cross the blood–brain barrier and activate glial cells, causing neuronal damage (Foster and McVey Neufeld, 2013). Studies indicate that the microbiota regulates the brain’s inflammatory response by modulating central and peripheral immune cells. Dysbiosis, characterized by an increase in pathogenic bacteria or a reduction in beneficial bacteria, may promote chronic inflammation that worsens cognitive and behavioral symptoms in dementia. Recent studies show that specific gut microbiota compositions, such as the abundance of Firmicutes and Verrucomicrobia, are associated with better cognitive functions, while increased levels of Bacteroidetes and Proteobacteria correlate with memory and cognitive deficits (Cryan and Dinan, 2012). These data support the role of the gut-brain axis, through which the microbiota regulates neuroinflammation, neurotransmitters, and blood–brain barrier integrity, directly influencing cognitive health (Aburto and Cryan, 2023; Loh et al., 2024). Various therapeutic strategies based on the gut microbiota are under development for the treatment or prevention of dementia. These include the use of probiotics, prebiotics, and fecal microbiota transplantation (FMT), which have shown promising results in animal models and preliminary human studies. Hoban et al. (2016) demonstrated that probiotics can positively influence behavior and cognition in animal models of dementia, suggesting a potential therapeutic approach to modulate the microbiota and improve neuropsychiatric symptoms.

This review identifies three convergent pathogenic pathways linking dysbiosis to cognitive decline: (1) metabolic starvation via reduced short-chain fatty acids (SCFA) and lactate producers (e.g., Faecalibacterium, Lactococcus phages); (2) systemic inflammation driven by P-glycoprotein dysfunction and LPS translocation; and (3) direct neurotoxicity via secondary bile acids and p-cresol.

## 2 Materials and methods

Given the breadth and complexity of the topic under investigation, this scoping review was conducted following the methodological framework proposed by Arksey and O’Malley (2005), later refined by Levac et al. (2010), and in accordance with the PRISMA-ScR (Preferred Reporting Items for Systematic Reviews and Meta-Analyses extension for Scoping Reviews) guidelines. This type of review was chosen because it allows for a systematic mapping of the existing literature, the identification of key thematic areas, the synthesis of available findings, and the detection of research gaps. The scoping review proved particularly suitable for exploring the role of the microbiota i cognitive decline, a multidisciplinary and rapidly evolving field characterized by significant methodological and conceptual heterogeneity among studies that led us to a final selection of 35 studies, deemed sufficient for a structured and thematically organized narrative synthesis.

### 2.1 Search strategy

In this scoping review, a structured methodology was used to evaluate research studies from 2014 to 2025, including all relevant data to ensure a comprehensive overview of the current evidence and minimize the risk of missing important information. We conducted an extensive literature search (between June 15, 2025, and July 15, 2025) through the databases PubMed, Web of Science, Cochrane Library, Embase, and Scopus, using the following keywords: (“dementia”[MeSH Terms] OR “dementia”[All Fields] OR “dementias”[All Fields] OR “dementia s”[All Fields]) AND (“cognition”[MeSH Terms] OR “cognition”[All Fields] OR “cognitions”[All Fields] OR “cognitive”[All Fields] OR “cognitively”[All Fields] OR “cognitives”[All Fields]) AND (“gut”[Journal] OR “gut”[All Fields]) AND (“gastrointestinal microbiome”[MeSH Terms] OR (“gastrointestinal”[All Fields] AND “microbiome”[All Fields]) OR “gastrointestinal microbiome”[All Fields] OR (“intestinal”[All Fields] AND “microbiome”[All Fields]) OR “intestinal microbiome”[AllFields]) AND (“microbiota”[MeSH Terms] OR “microbiota”[All Fields] OR “microbiotas”[All Fields] OR “microbiota s”[All Fields] OR “microbiotae”[All Fields]). The search strategy employed 5 core thematic blocks (Dementia, Cognition, Gut, Microbiome, Microbiota). To ensure reproducibility, we used specific MeSH terms combined with “All Fields” descriptors, resulting in a comprehensive string. Discrepancies in study selection were quantified using the Cohen’s kappa statistic, which yielded a value of 0.82, indicating substantial inter-rater reliability. These databases were carefully selected to cover a broad range of peer-reviewed literature relevant to this review. Using them helped maximize the completeness and validity of our search, reduce the risk of missing important studies, and ensure inclusion of diverse, high-quality evidence on the microbiota’s role in cognitive decline in dementia.

### 2.2 Inclusion criteria

This scoping review examined the relationship between gut microbiota and cognitive decline in dementia through a systematic search of six major databases: PubMed, Web of Science, Cochrane Library, Embase, and Scopus. Included were full-text English studies published between 2015 and 2025, focusing on human research that explicitly investigated the association between gut microbiota and dementia, with particular emphasis on AD and other cognitive disorders. The selection process followed clear inclusion criteria to ensure relevance and quality of evidence.

### 2.3 Exclusion criteria

All articles not specifically focused on the association between gut microbiota and dementia, regarding pathogenesis, disease progression, or potential therapies, were excluded. Studies on unrelated topics, such as general gut health or non-dementia neurological disorders, were also omitted. Additionally, studies without relevant outcomes, unclear target populations, or mixed samples not isolating dementia patients were excluded to maintain rigor. Study protocols, abstracts, editorials, letters, reviews, non-peer-reviewed articles, non-English papers, and those without full text were also excluded to ensure methodological quality.

### 2.4 Data extraction

**Figure 1:**
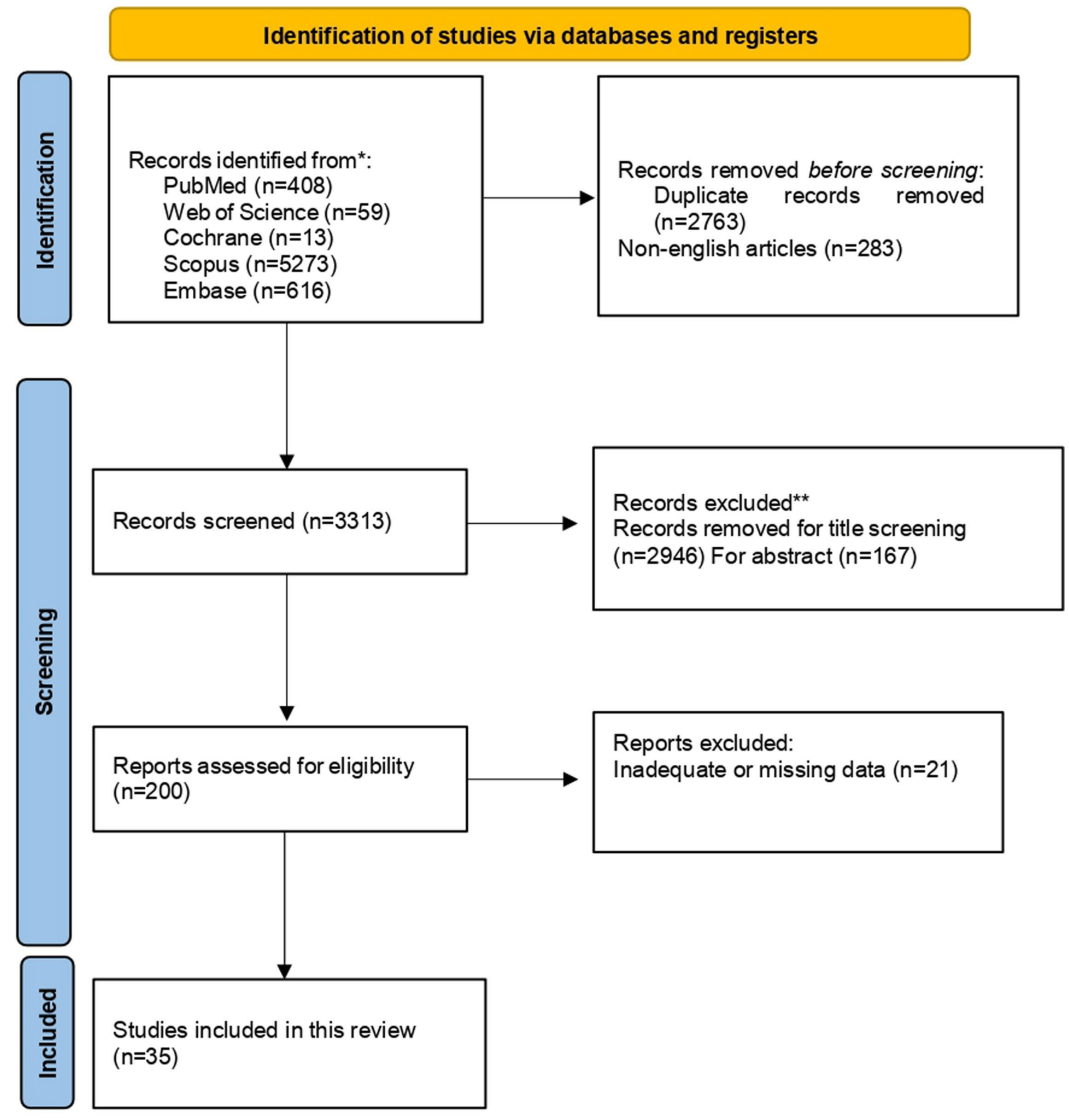
PRISMA flow diagram for research strategy. From Page et al. (2021). For more information, visit: http://www.prisma-statement.org/.

Two independent reviewers (GLC and GM) conducted the literature search, refining the strategy with keywords, Boolean operators, and MeSH terms. The study selection process was documented with a PRISMA flow diagram (Figure 1) (Haddaway et al., 2022), showing identification, screening, eligibility, and inclusion phases. Two other researchers (AC and SF) independently reviewed titles, abstracts, and full texts, extracted data, and cross-checked results to reduce bias. Disagreements were resolved by a third reviewer (AM). Reviewer agreement was assessed using the kappa statistic (Ryan et al., 2020), with values above 0.61 considered indicative of substantial inter-rater reliability. Datacollection and organization were supported by Microsoft Excel, which enabled efficient management of study attributes, risk of bias assessments, and outcome data. Customized extraction sheets and built- in features such as filtering and tagging facilitated consistency and helped resolve discrepancies. The relevance of the selected articles was then evaluated and summarized, highlighting key themes based on the inclusion and exclusion criteria.

This scoping review was registered on the Open Science Framework (OSF) under the following DOI: 10.17605/OSF.IO/CHQRG.

### 2.5 Synthesis of results

Given the variety of studies and factors involved in the relationship between microbiota and cognitive disorders in dementia, the data were analyzed using a narrative approach. This allowed highlighting the main themes and differences across research, providing a comprehensive overview. A multidisciplinary team ensured an objective interpretation of the results through meetings and discussions, reducing bias and ensuring consistency in the analysis.

## 3 Results

A comprehensive literature search was conducted across six electronic databases, initially retrieving 6,359 records. After removing 2,763 duplicates and 283 non-English articles, 3,313 records remained for title and abstract screening. During this phase, 2,946 articles were excluded based on titles, and 167 more after abstract review, leaving 200 articles for full-text retrieval. Despite efforts such as contacting authors and consulting libraries, 21 articles could not be accessed. The remaining 179 articles underwent detailed eligibility assessment, resulting in 35 studies meeting the inclusion criteria and included in this review (See PRISMA in Figure 1). The selected articles were categorized into five thematic macroareas, each representing a specific aspect of the relationship between gut microbiota and cognitive decline in dementia (Table 1). This classification allowed a systematic and coherent synthesis of the heterogeneous literature, facilitating comparison of findings and identification of common themes. This approach provided a clearer understanding of how microbiota alterations may influence cognitive impairment in dementia, improving the overall quality and focus of the review.

**Table 1:** Classification of selected articles into thematic macroareas.

Thematic macroareas	Author (year)	Title
Interventions on the microbiota to improve cognitive function	Akbari et al. (2016)	Effect of probiotic supplementation on cognitive function and metabolic status in AD: a randomized, double-blind and controlled trial
	Azuma et al. (2023)	Effect of continuous ingestion of bifidobacteria and dietary fiber on improvement in cognitive function: a randomized, double-blind, placebo-controlled trial
	Dumitrescu et al. (2022)	A microbial transporter of the dietary antioxidant ergothioneine
	Ghorbani et al. (2023)	A metagenomic study of gut viral markers in amyloid-positive AD patients
	Haran et al. (2019)	AD microbiome is associated with dysregulation of the anti-inflammatory P-glycoprotein pathway
	Hsu et al. (2023)	Efficacy of probiotic supplements on brain-derived neurotrophic factor, inflammatory biomarkers, oxidative stress and cognitive function in patients with Alzheimer’s dementia: a 12-week randomized, double-blind active-controlled study
	Jin et al. (2022)	Exploration of acupuncture therapy in the treatment of mild cognitive impairment based on the brain-gut axis theory
	Kobayashi et al. (2019)	Effects of *Bifidobacterium breve* A1 on the cognitive function of older adults with memory complaints: a randomised, double-blind, placebo-controlled trial
	Li et al. (2022)	Development of a gastrointestinal-myoelectrical-activity-based nomogram model for predicting the risk of mild cognitive impairment
	Liu et al. (2019)	Altered microbiomes distinguish AD from amnestic mild cognitive impairment and health in a Chinese cohort
	Ma et al. (2023)	Association between bowel movement pattern and cognitive function: prospective cohort study and a metagenomic analysis of the gut microbiome
	Oluwagbemigun et al. (2024)	An investigation into the relationship of circulating gut microbiome molecules and inflammatory markers with the risk of incident dementia in later life
	Saji et al. (2019)	Analysis of the relationship between the gut microbiome and dementia: a cross-sectional study conducted in Japan
	Sim et al. (2021)	Assessment of small intestinal bacterial overgrowth in AD
	Verdi et al. (2018)	An investigation into physical frailty as a link between the gut microbiome and cognitive health
	Yang et al. (2023)	Dysregulation of *Ruminococcaceae* and *Megamonas* could be predictive markers for rapid progression of mild cognitive impairment
Microbiota profiling and correlations with cognitive decline	Chen et al. (2022)	A comparison of the composition and functions of the oral and gut microbiotas in Alzheimer’s patients
	Chen et al. (2024)	A mediation analysis of the role of total free fatty acids on pertinence of gut microbiota composition and cognitive function in late life depression
	Khedr et al. (2022)	Alteration of gut microbiota in AD and their relation to the cognitive impairment
	Kim et al. (2024)	Alterations in gut microbiota and their correlation with brain beta-amyloid burden measured by 18F-florbetaben pet in mild cognitive impairment due to AD
	Marizzoni et al. (2023)	A peripheral signature of AD featuring microbiota-gut-brain axis markers
	Sheng et al. (2021)	Altered gut microbiota in adults with subjective cognitive decline: the SILCODE study
	Sritana and Phungpinij (2024)	Analysis of oral microbiota in elderly Thai patients with ad and mild cognitive impairment
	Wanapaisan et al. (2022)	Association between gut microbiota with mild cognitive Impairment and AD in a Thai population
	Yin et al. (2025)	Discovery of the microbiota-gut-brain axis mechanisms of acupuncture for amnestic mild cognitive impairment based on multi-omics analyses: a pilot study
	Zhang et al. (2021)	Diet quality, gut microbiota, and microRNAs associated with mild cognitive impairment in middle-aged and elderly Chinese population
	Zhu et al. (2022)	Altered gut microbiota and its clinical relevance in mild cognitive impairment and AD: Shanghai aging study and shanghai memory study
Molecular and metabolic mechanisms of the gut–brain axis	James et al. (2024)	Abnormalities in gut virome signatures linked with cognitive impairment in older adults
	MahmoudianDehkordi et al. (2019)	Altered bile acid profile associates with cognitive impairment in Alzheimer's disease—an emerging role for gut microbiome
	Nho et al. (2019)	Altered bile acid profile in mild cognitive impairment and AD: Relationship to neuroimaging and CSF biomarkers
	Wu et al. (2021)	Altered gut microbial metabolites in amnestic mild cognitive impairment and AD: signals in host-microbe interplay
	Zhao et al. (2023)	Association between erythrocyte membrane fatty acids and gut bacteria in obesity-related cognitive dysfunction
Individual and environmental factors influencing the microbiota and cognitive function	Deng et al. (2024)	Association between environmental phthalates exposure and gut microbiota and metabolome in dementia with Lewy bodies
	Hou et al. (2021)	The APOE ε4 allele is associated with a higher abundance of Proteobacteria, suggesting a possible link between genetic predisposition and gut dysbiosis
	Klee et al. (2024)	Education as risk factor of mild cognitive impairment: the link to the gut microbiome

The included studies were grouped into five thematic macroareas, each representing a specific dimension of the association between gut microbiota alterations and cognitive decline in dementia.

### 3.1 Interventions on the microbiota to improve cognitive function

This section includes studies that have investigated the effectiveness of interventions aimed at modulating the gut microbiota as a strategy to prevent or slow cognitive decline, showing promising results in various clinical and subclinical populations. Among the most established approaches, probiotic supplementation has shown beneficial effects in patients with AD. Akbari et al. (2016) reported a 27.9% improvement in MMSE scores in patients with Alzheimer’s disease treated with Lactobacillus and Bifidobacterium, a result associated with reduced inflammation and oxidative stress as well as improved insulin sensitivity. In contrast, Hsu et al. (2023) identified molecular changes indicative of neuroprotection—such as increased BDNF and decreased IL-1β and SOD—without, however, detecting significant changes in cognitive performance. Overall, probiotic supplementation shows heterogeneous outcomes but provides important insights into the potential biological mechanisms underlying gut–brain axis modulation. This discrepancy indicates that metabolic and neurotrophic restoration may precede detectable behavioral recovery, or that the therapeutic window for cognitive reversal is strictly limited to early-stage pathology. Non pharmacological interventions such as acupuncture are emerging as promising strategies for modulating the gut–brain axis. Using a multi omics approach, Yin et al. demonstrated that acupuncture can influence specific microbial taxa and cognition related metabolites, suggesting a potential role in restoring metabolic balance. Jin et al. (2022) further proposed a theoretical framework situating acupuncture within the broader dynamics of the gut–brain axis. Overall, these findings highlight therapeutic avenues that complement probiotic supplementation. Diet also emerges as an important modulating factor within this context. Zhang et al. (2021) showed that a high-quality diet, associated with a microbiota rich in Faecalibacterium and Alistipes and favorable microRNA profiles, was linked to a lower risk of MCI. The predictive model developed by the authors, integrating fecal microbiota data, dietary profiles, and plasma microRNAs, demonstrated high accuracy (AUC = 0.91) in distinguishing MCI subjects from healthy controls. Behavioral indicators such as intestinal motility have also been associated with cognitive function. Ma et al. (2023) found that extreme bowel movement frequencies (very low or very high) were linked to poorer cognitive performance and an unfavorable microbial profile (Roseburia, Veillonella, *Ruminococcus gnavus*), supporting the hypothesis that intestinal regularity reflects the status of the gut–brain axis. Sim et al. (2021) explored the hypothesis that specific gastrointestinal conditions, such as Small Intestinal Bacterial Overgrowth (SIBO), may contribute to Alzheimer’s disease AD pathogenesis. Although no significant differences in SIBO prevalence were found between Alzheimer’s patients and controls, the study highlighted the limitations of breath testing, which measures bacterial quantity but not quality. The authors suggest that other gastrointestinal regions, such as the colon, may be more involved in neurodegeneration, through mechanisms like increased intestinal permeability, chronic inflammation, and translocation of bacterial fragments into the brain—phenomena already observed in other neurodegenerative diseases such as Parkinson’s. Overall, these studies converge in suggesting that microbiota modulation—through probiotics, diet, acupuncture, or other interventions—may represent a promising strategy for the prevention and treatment of cognitive decline, especially when applied early and in a personalized manner.

### 3.2 Microbiota profiling and correlations with cognitive decline

This section focuses on studies that have analyzed the composition of the gut and oral microbiota in individuals with AD, mild cognitive impairment (MCI), and subjective cognitive decline, revealing distinctive patterns compared to healthy controls and suggesting a potential diagnostic and prognostic role. Chen et al. (2022) compared the oral and gut microbiota in Alzheimer’s patients, observing parallel alterations in both compartments as the disease progressed. Specifically, an increase in Firmicutes and Fusobacteria and a decrease in Proteobacteria were associated with lower MMSE scores and reduced microbial metabolic pathways related to amino acids, energy, and vitamins. A consistent “loss-of-function” signature emerges across cohorts, particularly regarding butyrate synthesis. Kim et al. (2024) and Wanapaisan et al. (2022) independently observed that cognitive decline tracks with the depletion of butyrate producers such as Faecalibacterium, Roseburia, and Ruminococcus. This reduction correlates with increased amyloid burden, supporting the hypothesis that loss of epigenetic regulation by SCFAs accelerates neurodegeneration. Concurrently, a pro-inflammatory shift is evident. Khedr et al. (2022) and Liu et al. (2019) noted increases in Proteobacteria and Escherichia-Shigella, taxa known to drive systemic inflammation. Crucially, Haran et al. (2019) identified a direct mechanistic link: this dysbiosis is associated with reduced intestinal P-glycoprotein (P-gp) expression. Since P-gp acts as a critical efflux pump for xenobiotics, its dysfunction allows pro-inflammatory molecules to accumulate and cross the blood–brain barrier, directly linking gut permeability to brain pathology. In the oral compartment, Sritana and Phungpinij (2024) described a pathogenic shift toward biofilm-forming taxa like *Fusobacterium nucleatum*, suggesting the oral cavity acts as a reservoir for systemic inflammatory mediators distinct from the gut. Finally, Marizzoni et al. (2023) proposed the existence of a “peripheral signature” of Alzheimer’s diseaseAD, detectable in the blood and associated with the microbiota. Patients exhibited a pro-inflammatory microbiota, elevated levels of LPS, and vascular and neurodegenerative markers (Aβ, pTau, neurofilaments), suggesting that cognitive decline reflects a systemic interaction between the gut, immune system, and brain.

### 3.3 Molecular and metabolic mechanisms of the gut–brain axis

A growing body of evidence demonstates that the gut microbiota influences brain function not only through its composition but also via metabolites and molecular signals that modulate inflammation, neurotoxicity, and neuronal metabolism. Among the most studied metabolites are bile acids, whose alterations have been documented in patients with AD and MCI by MahmoudianDehkordi et al. (2019) and Nho et al. (2019). Changes in bile acid profiles, associated with cerebrospinal fluid biomarkers and neuroimaging data, point to a role of the microbiota in regulating systemic inflammation and neurodegeneration. For instance, MahmoudianDehkordi et al. (2019) demonstrated a significant shift in the bile acid profile of AD patients, characterized by significantly lower levels of primary bile acids (e.g., cholic acid) and higher levels of secondary, bacterially-produced bile acids, specifically deoxycholic acid (DCA) and its conjugated forms. This increased DCA:cholic acid ratio was strongly correlated with cognitive decline and atrophy in brain regions critical for memory. Lipids also play a key role. Chen et al. (2024) showed that in elderly patients with depression, the abundance of Akkermansia was positively associated with cognitive performance (MoCA), while plasma levels of free fatty acids (FFAs) were inversely correlated. The reduction of *Akkermansia muciniphila* is a hallmark of dysbiosis in cognitive impairment, as this taxon is vital for maintaining intestinal barrier integrity. Regarding lipid signaling, Zhao T. et al. (2023) identified that higher levels of n-6 polyunsaturated fatty acids (PUFAs), such as arachidonic acid (C20:4 n-6) in erythrocyte membranes, are positively correlated with pro-inflammatory taxa like Coriobacteriales_Incertae_Sedis and negatively associated with MoCA scores, suggesting that an imbalance in the n-6/n-3 ratio exacerbates neuroinflammation via the gut-brain axis. FFAs appeared to partially mediate the positive effect of the microbiota on cognition. Similarly, Zhao et al. (2022) identified elevated levels of p-cresol sulfate, a toxic metabolite derived from protein fermentation, in patients with AD. This compound was linked to reduced hippocampal volume and poorer memory performance. Microbial metabolites such as short-269 chain fatty acids (SCFAs) were examined by Wu et al. (2021), who found a reduction in beneficial 270 compounds and an increase in neurotoxic metabolites in patients with Alzheimer’s and amnestic MCI. Innovative contribution came from Dumitrescu et al. (2022), who identified a microbial transporter of the antioxidant ergothioneine, potentially involved in protecting the brain from oxidative stress. Oluwagbemigun et al. (2024) explored the role of circulating microbial metabolites and inflammatory markers in dementia risk. Notably, low levels of 5OH-IAA, a serotonin catabolite with neuroprotective properties, were associated with increased dementia risk. Interestingly, no significant correlations were found with systemic inflammatory markers, suggesting that the microbiota’s contribution may occur primarily through metabolic pathways. Innovative insights into brain energy metabolism have emerged from virome analysis. Ghorbani et al. (2023) observed a specific depletion of Lactococcus prophages in AD patients. The authors hypothesize that this loss impairs the bacterial production of lactate—a critical energy substrate for neurons—thereby contributing to the cerebral hypometabolism characteristic of dementia. James et al. (2024) further confirmed a shift toward lytic interactions and reduced energy metabolism pathways, suggesting that viral dysbiosis actively sabotages the brain’s metabolic supply chain. Overall, these findings highlight the microbiota’s influence on brain health via microbial composition, metabolites, lipids, and virome dynamics, offering potential for early diagnosis and targeted therapies in cognitive decline.

### 3.4 Individual and environmental factors influencing the microbiota and cognitive function

Beyond direct interventions and microbial composition, several studies have shown that external and individual factors can modulate the gut ecosystem, with potential repercussions on cognitive health. Among environmental factors, exposure to phthalates (PAEs)—chemicals widely used in plastic materials—has been associated with gut dysbiosis and metabolic alterations in patients with Lewy body dementia. Deng et al. (2024) quantified 10 urinary phthalate metabolites using liquid chromatography–tandem mass spectrometry (LC–MS/MS), with concentrations adjusted for urinary creatinine to account for dilution. Specifically, higher levels of metabolites such as mono-2-ethylhexyl phthalate (MEHP) and mono-isobutyl phthalate (MiBP) were linked to a depletion of beneficial Bifidobacterium and an enrichment of *Ruminococcus gnavus*, a profile that correlates with increased neurotoxicity markers in Lewy body dementia. Moreover, fecal microbiota transplantation (FMT) from exposed patients into mice confirmed a negative impact on memory and behavior, suggesting a toxin-mediated neurodegenerative mechanism involving the microbiota. On the genetic side, Hou et al. (2021) showed that carriers of the APOE-ε4 allele, the main genetic risk factor for Alzheimer’s diseaseAD, exhibited gut dysbiosis characterized by an increase in pro-inflammatory bacteria (Proteobacteria, Escherichia–Shigella) and a decrease in SCFA-producing bacteria (Anaerostipes, Megamonas), indicating a potential interaction between genetic predisposition and microbial imbalance in the pathogenesis of cognitive decline. Sociodemographic factors, such as educational level, also influence microbiota composition. In the study by Klee et al. (2024), educational attainment was operationalized as “years of formal schooling” (continuous variable). The mediation analysis was robustly adjusted for age, sex, BMI, smoking status, and physical activity, revealing that higher education exerts a protective effect on cognition by fostering a more diverse microbiota, specifically counteracting the age-related decline of taxa involved in SCFA production. In the predictive domain, Li et al. (2020) proposed a Gut Dysbiosis Index (GDI) based on ten key bacterial taxa, capable of distinguishing individuals with MCI from cognitively healthy controls with 78% accuracy. The GDI was correlated with both cognitive scores and systemic inflammatory markers, offering a potential tool for early screening. Finally, Yang et al. (2023) identified the dysregulation of specific microbial taxa—particularly Ruminococcaceae and Megamonas—as potential markers of rapid MCI progression, paving the way for microbiota-based prognostic tools. Overall, these studies highlight how gut microbiota health is shaped by a complex interplay of environmental, genetic, social, and physiological factors, all of which interact with the central nervous system and contribute to cognitive risk.

## 4 Discussion

This scoping review analyzed numerous studies on the role of the gut and oral microbiota in cognitive decline, highlighting a growing integration between clinical, molecular, and environmental approaches. The findings outline a complex framework in which the microbiota emerges as a key element in the pathophysiology of neurodegenerative diseases, although the direction and robustness of these associations vary across studies due to methodological and demographic heterogeneity. Several studies have shown that modulation of the microbiota through probiotics and synbiotics can improve cognitive function, particularly in individuals with AD or MCI (Akbari et al., 2016; Azuma et al., 2023). Innovative approaches such as acupuncture and personalized diets (Yin et al., 2025; Zhang et al., 2021), as well as dietary interventions like intermittent fasting and ketogenic diets (Mela et al., 2025), demonstrate neuroprotective effects through microbiota regulation and inflammation control. As evidenced in the literature, the growing interest in integrative strategies—including fecal transplantation, physical exercise, and mindfulness suggests a shift toward multimodal approaches, although differences in intervention duration, strain specificity, and outcome measures make cross-study comparisons difficult (Bhadoriya et al., 2025). Nutritional and probiotic interventions appear to improve cognitive function in individuals with MCI or Alzheimer’s by acting on inflammation, SCFA production, BDNF modulation, and epigenetic regulation via miRNAs (Fu et al., 2022). However, their effectiveness is highly dependent on baseline cognitive status and individual microbiota composition, limiting generalizability across populations (Zhao T. et al., 2023; Zhao Y. et al., 2023). Microbiota profiling has revealed distinctive patterns between cognitively healthy individuals and patients with Alzheimer’s or MCI, with recurrent alterations in microbial diversity and bacterial taxa. Specifically, a reduction in butyrate-producing bacteria (e.g., Faecalibacterium, Roseburia) and an increase in pro-inflammatory species (e.g., Proteobacteria, Escherichia–Shigella) have been associated with neurodegenerative biomarkers such as amyloid burden and hippocampal volume reduction. Moreover, the presence of peripheral microbial signatures (Marizzoni et al., 2023) and plasma panels derived from the microbiota (Liu et al., 2023) opens new avenues for the development of non-invasive diagnostic tools (Zhang et al., 2025). Several studies suggest that cognitive decline is associated with the loss of beneficial bacteria (SCFA producers) and the increase of pro-inflammatory taxa, correlating with clinical and neurobiological biomarkers of AD (Zhu et al., 2022). Thus, the gut and oral microbiota emerge as potential early biomarkers and therapeutic targets (Chen et al., 2022). At the molecular level, its metabolites—such as SCFAs, bile acids, and neurotoxic compounds—influence inflammation, neuroplasticity, and brain metabolism (Wu et al., 2021; MahmoudianDehkordi et al., 2019; Garg et al., 2024). These data, consistent with the literature, indicate that SCFAs in particular play a key role in epigenetic and synaptic regulation (Zhang et al., 2025), opening new perspectives for early diagnosis and monitoring of cognitive decline. Nevertheless, metabolite measurements differ substantially across platforms (serum vs. plasma vs. feces), limiting comparability. Another interesting finding from this review is that the gut virome is emerging as a relevant component in neurocognitive health (Ghorbani et al., 2023). Alterations in bacteriophages can modify microbial composition and the production of neuroprotective metabolites, contributing to neuroinflammation and cognitive decline (James et al., 2024). The virome thus emerges as a potential biomarker, although current evidence is preliminary and limited by small sample sizes and heterogeneous methodologies. As highlighted in the literature, even the mycobiome—although still poorly studied—may influence brain function, enriching the complexity of the gut ecosystem (Bhadoriya et al., 2025). Individual and environmental factors, such as exposure to phthalates (Deng et al., 2024), genetic predisposition (Hou et al., 2021), education level (Klee et al., 2024), and intestinal motility (Ma et al., 2023), significantly influence microbiota composition. These associations, however, may be partially confounded by unmeasured variables such as diet, medications, lifestyle, and socioeconomic context, reinforcing the concept of AD as a systemic condition. Indices such as the Gut Dysbiosis Index (Li et al., 2020) and specific taxa associated with MCI progression (Yang et al., 2023) suggest the potential use of the microbiota as both a biomarker and therapeutic target. The preclinical stages of cognitive decline represent a critical window for intervention, as several studies indicate that subtle but detectable changes in microbiota composition and its metabolites occur during this phase, preceding the onset of measurable cognitive deficits (Wu et al., 2021; Yang et al., 2023; Yin et al., 2025). These modifications constitute potential early biomarkers and targets for timely preventive strategies. However, the limited number of longitudinal cohorts restricts the ability to determine causality or temporal sequencing of microbiota alterations. Numerous environmental (e.g., diet, pollutants), genetic (e.g., APOE ε4), socio-educational (e.g., education level), and physiological (e.g., intestinal motility) factors influence microbiota composition and function, contributing to cognitive decline through inflammatory, metabolic, and neurovascular mechanisms (Zhang et al., 2021; Deng et al., 2024; Hou et al., 2021). Yet the relative contribution of each factor remains difficult to quantify due to variability in study design and reporting practices. Several studies reviewed (Akbari et al., 2016; Azuma et al., 2023; Hsu et al., 2023) have shown that probiotic supplementation, alone or combined with prebiotic fibers, can improve cognitive function in individuals with MCI or AD. These results have also been confirmed in healthy elderly populations, with benefits in memory, attention, and executive flexibility (Fekete et al., 2024). The proposed mechanisms include modulation of systemic inflammation, increased SCFA production, and BDNF regulation. In parallel, microbiota analysis has revealed significant alterations in individuals with cognitive impairment, including a reduction in beneficial taxa such as Faecalibacterium and Ruminococcaceae, and an increase in pro-inflammatory bacteria such as Proteobacteria and Escherichia–Shigella (Zhu et al., 2022; Wanapaisan et al., 2022; Grabrucker et al., 2023). Another emerging area concerns the oral microbiota, increasingly recognized as a potential early biomarker. Reviewed studies (Chen et al., 2022; Sritana and Phungpinij, 2024) have highlighted the role of pathogenic oral bacteria, such as *Fusobacterium nucleatum* and *Porphyromonas gingivalis*, in neuroinflammation and amyloid aggregation, as also confirmed by other literature data (Lim et al., 2017). Still, oral-microbiome methodologies vary substantially across studies. At the molecular level, microbial metabolites, particularly SCFAs like butyrate, play a neuroprotective role, while other compounds such as Trimethylamine N-oxide (TMAO) and toxic bile acids are associated with AD biomarkers (MahmoudianDehkordi et al., 2019; Nho et al., 2019; Wu et al., 2021; Kim et al., 2024).

**Figure 2:**
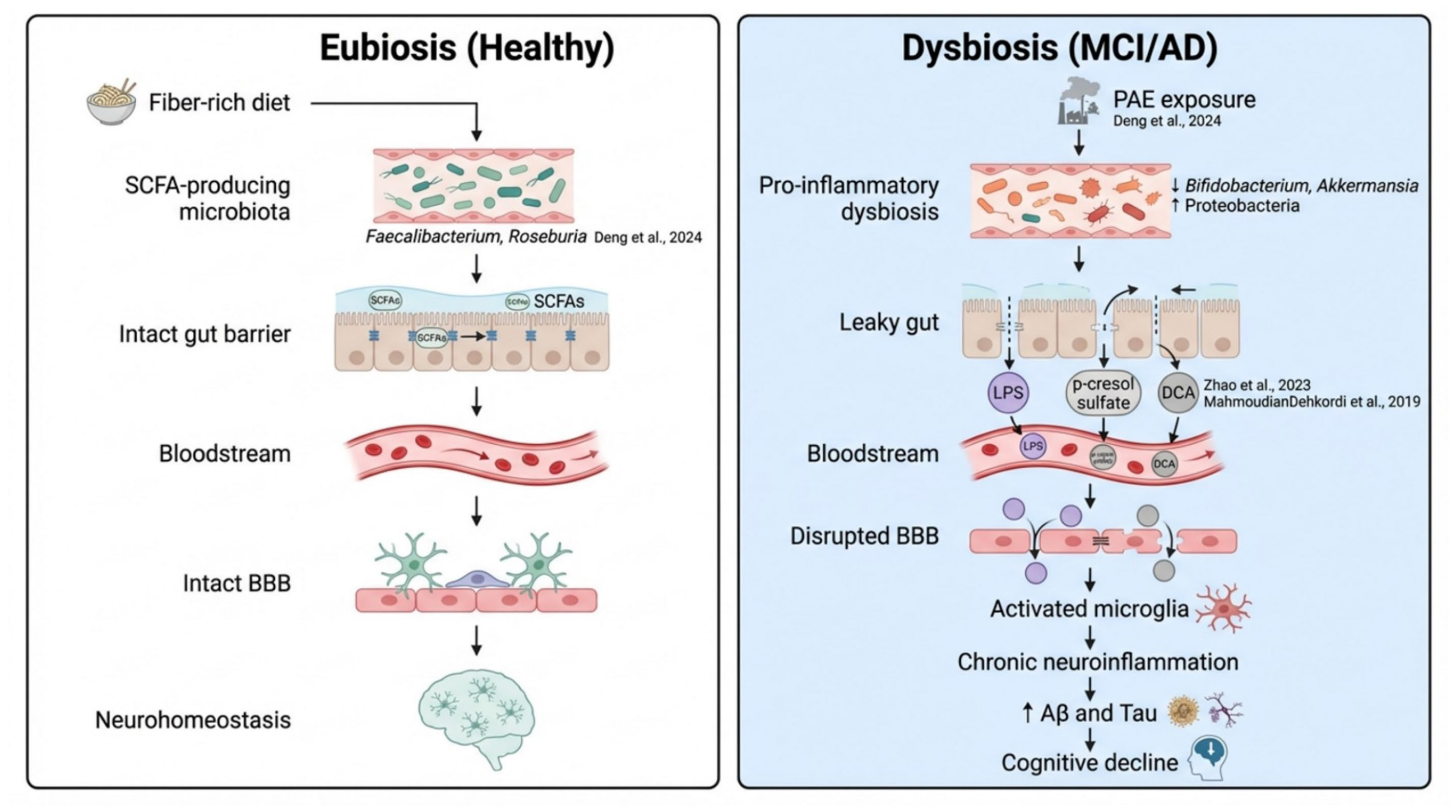
Proposed mechanistic pathways of the gut-brain axis in dementia progression. The figure contrasts physiological eubiosis (left) with the pathological state of MCI and AD (right). In eubiosis, a fiber-rich diet supports SCFA-producing taxa (*Faecalibacterium, Bifidobacterium*), maintaining intestinal barrier and blood–brain barrier (BBB) integrity. In the dysbiotic state, environmental stressors such as phthalate (PAE) exposure (Deng et al., 2024) trigger a loss of beneficial microbes and a bloom of pro-inflammatory taxa (*Proteobacteria*). This leads to a “leaky gut” and the systemic translocation of neurotoxic metabolites, including *p*-cresol sulfate (Zhao et al., 2023), lipopolysaccharides (LPS), and secondary bile acids like deoxycholic acid (DCA) (MahmoudianDehkordi et al., 2019). These mediators cross the compromised BBB, activating microglia and promoting amyloid-β deposition and neurodegeneration.

Finally, integrated and multi-omic approaches are revolutionizing the understanding of cognitive decline. Studies such as those by Yang et al. (2023) and Yin et al. (2025) have developed predictive models based on microbial, metabolic, and neuroimaging signatures capable of early identification of individuals at risk of progression from MCI to dementia. Nevertheless, differences in computational pipelines and lack of standardized analytical frameworks limit reproducibility. The reviewed evidence supports a model where cognitive decline is driven by a “double hit”: (1) the loss of neuroprotective metabolites (ergothioneine, SCFAs) and (2) the active production of neurotoxins (secondary bile acids, p-cresol). This shifts the therapeutic focus from simply “adding good bacteria” to restoring specific metabolic pathways. Taken together, while the existing literature highlights promising microbiota–brain interactions, these methodological limitations caution against overinterpretation and underscore the need for more rigorous, standardized, and longitudinal research frameworks. The complex interplay between gut dysbiosis, metabolite translocation, and neuroinflammatory pathways is mechanistically summarized in Figure 2.

## 5 Conclusion

This scoping review highlights the central role of the gut and oral microbiota in the pathophysiology of cognitive decline, reinforcing the hypothesis of the gut–brain axis in the progression of MCI and AD. Compositional alterations, including the loss of beneficial taxa and the increase of pro-inflammatory species, are associated with cognitive and neuroinflammatory dysfunctions. In this framework, the microbiota thus emerges as a non-invasive diagnostic biomarker and a modifiable therapeutic target. Interventions such as probiotics, synbiotics, dietary modifications, and multimodal approaches show promising effects, especially in the early stages of cognitive impairment. Furthermore, the integration of multi-omic strategies and predictive models opens new perspectives for precision medicine. However, the interpretation of these findings is limited by several factors. High methodological heterogeneity, geographical variability, and the lack of longitudinal studies substantially reduce the generalizability of current findings. Additional challenges include the variability of sequencing techniques, inconsistent metabolite-profiling methods, and limited control of confounding lifestyle and environmental factors. Ultimately, this review highlights that the microbiota–gut–brain axis is not just a biomarker of disease, but an active, modifiable driver of AD pathology via specific metabolic and immune checkpoints (e.g., P-gp modulation, bile acid metabolism), offering tangible targets for precision medicine.

## 6 Future directions

Future research should focus on longitudinal and multicenter studies conducted on large and diverse cohorts to validate microbial and metabolic signatures as predictive biomarkers of progression from MCI to AD. Randomized controlled trials will be necessary to evaluate personalized microbiota-targeted interventions, including probiotics, dietary strategies, and fecal microbiota transplantation. The integration of multi-omic approaches (metagenomics, metabolomics, epigenomics, neuroimaging) is increasingly recognized as crucial to better understand microbiota–brain interactions. Additionally, further exploration of the oral microbiome, virome, and mycobiome is remains essential, along with the promotion of interdisciplinary approaches to develop precision medicine strategies. Importantly, standardized protocols for sample collection, sequencing, metabolite quantification, and data reporting will be necessary to improve comparability across studies and strengthen the translational potential of microbiota-based biomarkers.

## 7 Strengths and limitations

The main strength of this scoping review lies in its systematic and multidimensional approach, which enabled an integrated analysis of the microbiota’s role in cognitive decline. The inclusion of clinical, observational, and experimental studies, organized into five thematic macro-areas, provided a broad and up-to-date overview, with particular attention to emerging fields such as the gut virome and oral microbiome. However, the substantial methodological heterogeneity across studies—ranging from sequencing platforms to metabolite-profiling techniques-, the predominance of observational and cross-sectional designs, and the limited integration of multi-omic data restrict significantly limit differences in dietary and environmental exposures, and inconsistent control of confounding factors further constrain generalizability. The absence of longitudinal trajectories prevents the identification of temporal dynamics and early inflection points in microbiota-related changes. Despite these limitations, the review offers a solid foundation to guide future research and inform the development of more rigorous, personalized therapeutic strategies grounded in microbiota-driven mechanisms rather than purely taxonomic changes.

## References

[ref1] Aburto M. R. Cryan J. F. (2023). Gastrointestinal and brain barriers: unlocking gates of communication across the microbiota–gut–brain axis. Nat. Rev. Gastroenterol. Hepatol. 20, 641–656. doi: 10.1038/s41575-023-00890-0, 38355758

[ref2] Akbari E. Asemi Z. Daneshvar Kakhaki R. Bahmani F. Kouchaki E. Tamtaji O. R. . (2016). Effect of probiotic supplementation on cognitive function and metabolic status in Alzheimer's disease: a randomized, double-blind and controlled trial. Front. Aging Neurosci. 8:256. doi: 10.3389/fnagi.2016.00256, 27891089 PMC5105117

[ref3] Arksey H. O’Malley L. (2005). Scoping studies: towards a methodological framework. Int. J. Soc. Res. Methodol. 8, 19–32. doi: 10.1080/1364557032000119616

[ref4] Azuma N. Mawatari T. Saito Y. Tsukamoto M. Sampei M. Iwama Y. (2023). Effect of continuous ingestion of Bifidobacteria and dietary fiber on improvement in cognitive function: a randomized, double-blind, placebo-controlled trial. Nutrients 15:4175. doi: 10.3390/nu15194175, 37836458 PMC10574581

[ref5] Bhadoriya P. Jatley A. Singh A. Mehrotra R. Jain M. Mohammed A. . (2025). Exploring gut microbiota's influence on cognitive health and neurodegenerative disorders: mechanistic insights and therapeutic approaches. Discover Immun. 2:3. doi: 10.1007/s44368-025-00010-x

[ref6] Cattaneo A. Cattane N. Galluzzi S. Provasi S. Lopizzo N. Festari C. . (2017). Altered gut microbiota in Alzheimer's disease patients. Sci. Rep. 7:11522.28912589

[ref7] Cerejeira J. Lagarto L. Mukaetova-Ladinska E. B. (2012). Behavioral and psychological symptoms of dementia. Front. Neurol. 3:73. doi: 10.3389/fneur.2012.00073, 22586419 PMC3345875

[ref8] Chen Y. Li J. Le D. Zhang Y. Liao Z. (2024). A mediation analysis of the role of total free fatty acids on pertinence of gut microbiota composition and cognitive function in late life depression. Lipids Health Dis. 23:64. doi: 10.1186/s12944-024-02056-6, 38424549 PMC10903004

[ref9] Chen L. Xu X. Wu X. Cao H. Li X. Hou Z. . (2022). A comparison of the composition and functions of the oral and gut microbiotas in Alzheimer's patients. Front. Cell. Infect. Microbiol. 12:942460. doi: 10.3389/fcimb.2022.942460, 36093178 PMC9448892

[ref10] Cryan J. F. Dinan T. G. (2012). Mind-altering microorganisms: the impact of the gut microbiota on brain and behaviour. Nat. Rev. Neurosci. 13, 701–712. doi: 10.1038/nrn3346, 22968153

[ref11] Cryan J. F. O'Mahony S. M. (2011). The microbiome-gut-brain axis: from bowel to brain. Nat. Rev. Gastroenterol. Hepatol. 9, 590–596.

[ref12] Deng Z. Li L. Jing Z. Luo X. Yu F. Zeng W. . (2024). Association between environmental phthalates exposure and gut microbiota and metabolome in dementia with Lewy bodies. Environ. Int. 190:108806. doi: 10.1016/j.envint.2024.108806, 38908272

[ref13] Dumitrescu D. G. Gordon E. M. Kovalyova Y. Seminara A. B. Duncan-Lowey B. Forster E. R. . (2022). A microbial transporter of the dietary antioxidant ergothioneine. Cell 185, 4526–4540.e18. doi: 10.1016/j.cell.2022.10.008, 36347253 PMC9691600

[ref14] Fekete M. Lehoczki A. Major D. Fazekas-Pongor V. Csípő T. Tarantini S. . (2024). Exploring the influence of gut–brain axis modulation on cognitive health: a comprehensive review of prebiotics, probiotics, and symbiotics. Nutrients 16:789. doi: 10.3390/nu16060789, 38542700 PMC10975805

[ref15] Foster J. A. McVey Neufeld K.-A. (2013). Gut–brain axis: how the microbiome influences anxiety and depression. Trends Neurosci. 36, 305–312. doi: 10.1016/j.tins.2013.01.005, 23384445

[ref16] Fu J. Tan L. J. Lee J. E. Shin S. (2022). Association between the mediterranean diet and cognitive health among healthy adults: a systematic review and meta-analysis. Front. Nutr. 9:946361. doi: 10.3389/fnut.2022.946361, 35967772 PMC9372716

[ref17] Garg M. Karpinski M. Matelska D. Middleton L. Burren O. S. Hu F. . (2024). Disease prediction with multi-omics and biomarkers empowers case-control genetic discoveries in the UK biobank. Nat. Genet. 56, 1821–1831. doi: 10.1038/s41588-024-01898-1, 39261665 PMC11390475

[ref18] Ghorbani M. Ferreira D. Maioli S. (2023). A metagenomic study of gut viral markers in amyloid-positive Alzheimer's disease patients. Alzheimer's Res. Ther. 15:141. doi: 10.1186/s13195-023-01285-8, 37608325 PMC10464408

[ref9001] Grabrucker AM Subramaniam M van de Sande T Schmidt M. (2023). The gut microbiome in neurodegeneration: emerging role in Alzheimer’s disease. Int J Mol Sci. 24:4567. doi: 10.3390/ijms24054567 36901998 PMC10003459

[ref19] Haddaway N. R. Page M. J. Pritchard C. C. McGuinness L. A. (2022). PRISMA2020: an R package and shiny app for producing PRISMA 2020-compliant flow diagrams, with interactivity for optimised digital transparency and open synthesis. Campbell Syst. Rev. 18:e1230. doi: 10.1002/cl2.1230, 36911350 PMC8958186

[ref20] Haran J. P. Bhattarai S. K. Foley S. E. Dutta P. Ward D. V. Bucci V. . (2019). Alzheimer's disease microbiome is associated with dysregulation of the anti-inflammatory P-glycoprotein pathway. MBio 10:e00632-19. doi: 10.1128/mBio.00632-19, 31064831 PMC6509190

[ref21] Hoban A. E. Stilling R. M. Ryan F. J. Shanahan F. Dinan T. G. Cryan J. F. (2016). The microbiome regulates amygdala-dependent fear recall. Mol. Psychiatry 21, 46–53. 10.1038/mp.2017.100 PMC5984090 28507320

[ref22] Hou M. Xu G. Ran M. Luo W. Wang H. (2021). APOE-ε4 carrier status and gut microbiota Dysbiosis in patients with Alzheimer disease. Front. Neurosci. 15:619051. doi: 10.3389/fnins.2021.619051, 33732104 PMC7959830

[ref23] Hsu Y. C. Huang Y. Y. Tsai S. Y. Kuo Y. W. Lin J. H. Ho H. H. . (2023). Efficacy of probiotic supplements on brain-derived neurotrophic factor, inflammatory biomarkers, oxidative stress and cognitive function in patients with Alzheimer's dementia: a 12-week randomized, double-blind active-controlled study. Nutrients 16:16. doi: 10.3390/nu16010016, 38201846 PMC10780998

[ref24] James A. S. Adil N. A. Goltz D. Tangudu D. Chaudhari D. S. Shukla R. . (2024). Abnormalities in gut virome signatures linked with cognitive impairment in older adults. Gut Microbes 16:2431648. doi: 10.1080/19490976.2024.2431648, 39676708 PMC11651276

[ref25] Javaid S. Giebel C. Khan M. Hashim M. (2021). Epidemiology of Alzheimer’s disease and other dementias: rising global burden and forecasted trends. F1000Res 10:425. doi: 10.12688/F1000RESEARCH.50786.1

[ref26] Jin Y. Hu F. Zhu J. (2022). Exploration of acupuncture therapy in the treatment of mild cognitive impairment based on the brain-gut axis theory. Front. Hum. Neurosci. 16:891411. doi: 10.3389/fnhum.2022.891411, 36204718 PMC9531719

[ref27] Khedr E. M. Omeran N. Karam-Allah Ramadan H. Ahmed G. K. Abdelwarith A. M. (2022). Alteration of gut microbiota in Alzheimer's disease and their relation to the cognitive impairment. J. Alzheimer's Dis 88, 1103–1114. doi: 10.3233/JAD-220176, 35754271

[ref28] Kim S. H. Jazwinski S. M. (2019). Dysbiosis of gut microbiota and its potential implications in Alzheimer's disease. J. Alzheimer's Dis 67, 1–12.30452418

[ref29] Kim G. H. Kim B. R. Yoon H. J. Jeong J. H. (2024). Alterations in gut microbiota and their correlation with brain Beta-amyloid burden measured by 18F-Florbetaben PET in mild cognitive impairment due to Alzheimer's disease. J. Clin. Med. 13:1944. doi: 10.3390/jcm13071944, 38610709 PMC11012963

[ref30] Klee M. Aho V. T. E. May P. Heintz-Buschart A. Landoulsi Z. Jónsdóttir S. R. . (2024). Education as risk factor of mild cognitive impairment: the link to the gut microbiome. J. Prev Alzheimers Dis. 11, 759–768. doi: 10.14283/jpad.2024.19, 38706292 PMC11060993

[ref31] Kobayashi Y. Kuhara T. Oki M. Xiao J. Z. (2019). Effects of *Bifidobacterium breve* A1 on the cognitive function of older adults with memory complaints: a randomised, double-blind, placebo-controlled trial. Benef. Microbes 10, 511–520. doi: 10.3920/BM2018.0170, 31090457

[ref32] Levac D. Colquhoun H. O’Brien K. K. (2010). Scoping studies: advancing the methodology. Implement. Sci. 5:69. doi: 10.1186/1748-5908-5-69, 20854677 PMC2954944

[ref9003] Li B He Y Ma J Huang P Du J Cao L . (2020). Mild cognitive impairment has similar alterations as Alzheimer’s disease in gut microbiota. Alzheimers Dement. 16, 332–345. doi: 10.1002/alz.12031 31434623

[ref33] Li B. Ji S. Peng A. Yang N. Zhao X. Feng P. . (2022). Development of a gastrointestinal-Myoelectrical-activity-based nomogram model for predicting the risk of mild cognitive impairment. Biomolecules 12:1861. doi: 10.3390/biom12121861, 36551289 PMC9775682

[ref34] Liang S. Wu X. Jin F. (2018). The gut-brain connection: a new frontier in the development of therapeutic strategies for Alzheimer's disease. J. Alzheimer's Dis 64, 49–56.29865051

[ref35] Lim Y. Totsika M. Morrison M. Punyadeera C. (2017). Oral microbiome: a new biomarker reservoir for Oral and oropharyngeal cancers. Theranostics 7, 4313–4321. doi: 10.7150/thno.21804, 29158828 PMC5695015

[ref36] Liu P. Wu L. Peng G. Han Y. Tang R. Ge J. . (2019). Altered microbiomes distinguish Alzheimer's disease from amnestic mild cognitive impairment and health in a Chinese cohort. Brain Behav. Immun. 80, 633–643. doi: 10.1016/j.bbi.2019.05.008, 31063846

[ref9004] Liu S Gao J Zhu M Liu K Zhang HL. (2023). Gut microbiota and dysbiosis in Alzheimer’s disease: implications for pathogenesis and therapy. Front Cell Infect Microbiol. 13:1173245. doi: 10.3389/fcimb.2023.1173245

[ref37] Loh J. S. Mak W. Q. Tan L. K. S. Ng C. X. Chan H. H. Yeow S. H. . (2024). Microbiota–gut–brain axis and its therapeutic applications in neurodegenerative diseases. Signal Transduct. Target. Ther. 9:37. doi: 10.1038/s41392-024-01743-1, 38360862 PMC10869798

[ref38] Ma C. Li Y. Mei Z. Yuan C. Kang J. H. Grodstein F. . (2023). Association between bowel movement pattern and cognitive function: prospective cohort study and a metagenomic analysis of the gut microbiome. Neurology 101, e2014–e2025. doi: 10.1212/WNL.0000000000207849, 37775319 PMC10662989

[ref39] MahmoudianDehkordi S. Arnold M. Nho K. Ahmad S. Jia W. Xie G. . (2019). Altered bile acid profile associates with cognitive impairment in Alzheimer's disease—an emerging role for gut microbiome. Alzheimers Dement. 15, 76–92. doi: 10.1016/j.jalz.2018.07.217, 30337151 PMC6487485

[ref40] Maier E. Anderson R. C. Roy N. C. (2017). The role of the gut microbiota in the pathogenesis of Alzheimer's disease. J. Neural Transm. 124, 367–375.

[ref41] Marizzoni M. Mirabelli P. Mombelli E. Coppola L. Festari C. Lopizzo N. . (2023). A peripheral signature of Alzheimer's disease featuring microbiota-gut-brain axis markers. Alzheimer's Res. Ther. 15:101. doi: 10.1186/s13195-023-01218-5, 37254223 PMC10230724

[ref42] Mela V. Heras V. Iesmantaite M. García-Martín M. L. Bernal M. Posligua-García J. D. . (2025). Microbiota fasting-related changes ameliorate cognitive decline in obesity and boost *ex vivo* microglial function through the gut-brain axis. Gut 74:335353. doi: 10.1136/gutjnl-2025-335353, 40335161 PMC12917722

[ref43] Nho K. Kueider-Paisley A. MahmoudianDehkordi S. Arnold M. Risacher S. L. Louie G. . (2019). Altered bile acid profile in mild cognitive impairment and Alzheimer's disease: relationship to neuroimaging and CSF biomarkers. Alzheimers Dement. 15, 232–244. doi: 10.1016/j.jalz.2018.08.012, 30337152 PMC6454538

[ref44] Oluwagbemigun K. Anesi A. Vrhovsek U. Mattivi F. Martino Adami P. Pentzek M. . (2024). An investigation into the relationship of circulating gut microbiome molecules and inflammatory markers with the risk of incident dementia in later life. Mol. Neurobiol. 61, 9776–9793. doi: 10.1007/s12035-023-03513-6, 37605096 PMC11584436

[ref45] Page M. J. McKenzie J. E. Bossuyt P. M. Boutron I. Hoffmann T. C. Mulrow C. D. . (2021). The PRISMA 2020 statement: an updated guideline for reporting systematic reviews. BMJ 372:n71. doi: 10.1136/bmj.n71 33782057 PMC8005924

[ref46] Ryan M. M. Spotnitz W. D. Gillen D. L. (2020). Variance estimation for the kappa statistic in the presence of clustered data and heterogeneous observations. Stat. Med. 39, 1941–1951. doi: 10.1002/sim.8522, 32180248

[ref47] Saji N. Niida S. Murotani K. Hisada T. Tsuduki T. Sugimoto T. . (2019). Analysis of the relationship between the gut microbiome and dementia: a cross-sectional study conducted in Japan. Sci. Rep. 9:1008. doi: 10.1038/s41598-018-38218-7, 30700769 PMC6353871

[ref48] Sgambato J. O’Mahony S. (2020). The gut microbiome and neurodegeneration: exploring the links with Alzheimer's disease. Curr. Neuropharmacol. 18, 859–872.

[ref49] Sheng C. Lin L. Lin H. Wang X. Han Y. Liu S. L. (2021). Altered gut microbiota in adults with subjective cognitive decline: the SILCODE study. J. Alzheimer's Dis 82, 513–526. doi: 10.3233/JAD-210259, 34024839

[ref50] Sim J. Wang Y. T. Mamun K. Tay S. Y. Doshi K. Hameed S. . (2021). Assessment of small intestinal bacterial overgrowth in Alzheimer's disease. Acta Neurol. Taiwanica 30, 102–107. 34841505

[ref51] Sritana N. Phungpinij A. (2024). Analysis of oral microbiota in elderly Thai patients with Alzheimer's disease and mild cognitive impairment. Int. J. Environ. Res. Public Health 21:1242. doi: 10.3390/ijerph21091242, 39338124 PMC11431138

[ref52] Takahashi R. Nagao T. Gouras G. (2017). Plaque formation and the intraneuronal accumulation of β-amyloid in Alzheimer's disease. Pathol. Int. 67, 185–193. doi: 10.1111/pin.12520, 28261941

[ref54] Verdi S. Jackson M. A. Beaumont M. Bowyer R. C. E. Bell J. T. Spector T. D. . (2018). An investigation into physical frailty as a link between the gut microbiome and cognitive health. Front. Aging Neurosci. 10:398. doi: 10.3389/fnagi.2018.00398, 30564113 PMC6288358

[ref9002] Vogt NM Kerby RL Dill-McFarland KA Harding SJ Merluzzi AP Johnson SC . (2017). Gut microbiome alterations in Alzheimer’s disease. Sci Rep. 7:13537. doi: 10.1038/s41598-017-13601-y 29051531 PMC5648830

[ref55] Wanapaisan P. Chuansangeam M. Nopnipa S. Mathuranyanon R. Nonthabenjawan N. Ngamsombat C. . (2022). Association between gut microbiota with mild cognitive impairment and Alzheimer's disease in a Thai population. Neurodegener Dis 22, 43–54. doi: 10.1159/000526947, 36070704

[ref56] Wu L. Han Y. Zheng Z. Peng G. Liu P. Yue S. . (2021). Altered gut microbial metabolites in amnestic mild cognitive impairment and Alzheimer's disease: signals in host-microbe interplay. Nutrients 13:228. doi: 10.3390/nu13010228, 33466861 PMC7829997

[ref57] Yang J. Wang L. Liu H. Xu H. Liu F. Song H. . (2023). Dysregulation of Ruminococcaceae and Megamonas could be predictive markers for rapid progression of mild cognitive impairment. Microb. Pathog. 183:106272. doi: 10.1016/j.micpath.2023.106272, 37543169

[ref58] Yin Z. H. Bao Q. N. Li Y. Q. Liu Y. W. Wang Z. Q. Ye F. . (2025). Discovery of the microbiota-gut-brain axis mechanisms of acupuncture for amnestic mild cognitive impairment based on multi-omics analyses: a pilot study. Complement. Ther. Med. 88:103118. doi: 10.1016/j.ctim.2024.103118, 39667708

[ref59] Zhang Q. Gao Z. Deng Y. Xu X. Sun W. Liu R. . (2025). Current status and trends in the study of intestinal flora in cognitive disorders: a bibliometric and visual analysis. Front. Microbiol. 16:1577597. doi: 10.3389/fmicb.2025.1577597, 40497058 PMC12148866

[ref60] Zhang X. Wang Y. Liu W. Wang T. Wang L. Hao L. . (2021). Diet quality, gut microbiota, and microRNAs associated with mild cognitive impairment in middle-aged and elderly Chinese population. Am. J. Clin. Nutr. 114, 429–440. doi: 10.1093/ajcn/nqab078, 33871591

[ref61] Zhao T. Huang H. Li J. Shen J. Zhou C. Xiao R. . (2023). Association between erythrocyte membrane fatty acids and gut bacteria in obesity-related cognitive dysfunction. AMB Express 13:148. doi: 10.1186/s13568-023-01655-3, 38123761 PMC10733235

[ref62] Zhao Y. Jaber V. Lukiw W. J. (2017). The microbiome and Alzheimer's disease: the gut-brain axis. Curr. Alzheimer Res. 14, 776–784.

[ref9005] Zhao Y Lukiw WJ. (2022). Microbiome-generated amyloid and potential impact on Alzheimer’s disease. J Alzheimers Dis. 85, 1–14. doi: 10.3233/JAD-215123 26097896 PMC4469284

[ref63] Zhao Y. Lei Y. Ning H. Zhang Y. Chen G. Wang C. . (2023). PGF2α facilitates pathological retinal angiogenesis by modulating endothelial FOS-driven ELR+ CXC chemokine expression. EMBO Mol. Med. 15:e16373. doi: 10.15252/emmm.202216373, 36511116 PMC9832840

[ref64] Zhu Z. Ma X. Wu J. Xiao Z. Wu W. Ding S. . (2022). Altered gut microbiota and its clinical relevance in mild cognitive impairment and Alzheimer's disease: Shanghai aging study and Shanghai memory study. Nutrients 14:3959. doi: 10.3390/nu14193959, 36235612 PMC9570603

